# Trends in Plants Phytochemistry and Bioactivity Analysis

**DOI:** 10.3390/plants13223173

**Published:** 2024-11-12

**Authors:** Nijole Savickiene, Lina Raudone

**Affiliations:** 1 Department of Pharmacognosy, Lithuanian University of Health Sciences, Sukileliu Av. 13, 50162 Kaunas, Lithuania; 2 Laboratory of Biopharmaceutical Research, Institute of Pharmaceutical Technologies, Lithuanian University of Health Sciences, Sukileliu Av. 13, 50162 Kaunas, Lithuania

Biologically active compounds, derived from various natural sources, have garnered significant attention due to their potential therapeutic applications. These compounds, which include essential oils, phenolics, flavonoids, and other phytochemicals, exhibit a wide range of biological activities such as antioxidant, antimicrobial, anticancer, and anti-inflammatory effects. This article explores recent studies highlighting the diverse applications and benefits of biologically active compounds extracted from plants and other natural sources. The studies outlined here highlight the therapeutic potential of natural compounds, suggesting applications in medicine, food preservation, and agriculture.

The global use of herbal medicinal products continues to grow. Extensive research is being performed on plant materials and the development of their preparations for the management of chronic diseases. Plant raw material and the quality of products made from it must meet the requirements of safety and stability efficiency, and bioactive compounds are the key determinants. The studies outlined here underscore the importance of exploring natural compounds for several reasons. Many of the natural extracts and compounds show promising therapeutic properties, offering potential alternatives to synthetic drugs in treating diseases such as cancer, diabetes, and obesity [1,2,3].

Overall, these investigations highlight the vast potential of plant-based compounds in enhancing health and wellness while also promoting sustainable and environmentally friendly practices. The presented publication is a compilation of various scientific studies, each focusing on different natural compounds and their potential health benefits. The following is a step-by-step synthesis of the main findings of these studies.

Ouakouak et al. investigated the chemical composition and biological activities of essential oils (EOs) extracted from *Thymus algeriensis* (Contribution 1). EOs were extracted using three methods—hydrodistillation, steam distillation, and microwave-assisted distillation—during different flowering stages. A total of 85 compounds were identified, with oxygenated monoterpenes being predominant, particularly camphor, borneol, and 1,8-cineole. The EOs demonstrated significant antitumor activity against colon cancer cells (HCT116) and moderate activity against hepatocellular carcinoma cells (HePG2). Additionally, the oils exhibited notable antimicrobial properties against various bacterial and fungal strains. The findings of this study highlight the potential therapeutic applications of *Thymus algeriensis* essential oils in medicine and food preservation.

The study by Niaz et al. on *Tamarix dioica* revealed that the ethyl acetate fraction of its extract exhibited strong antioxidant and α-glucosidase inhibitory activities, supporting its traditional use in managing diabetes (Contribution 2). The researchers fractionated the methanolic extract of *T. dioica* into several solvent fractions (n-hexane, ethyl acetate, chloroform, and n-butanol) and assessed their antioxidant and α-glucosidase inhibitory activities. The ethyl acetate fraction emerged as the most effective, exhibiting a DPPH scavenging activity of 84.44% and an α-glucosidase inhibition IC_50_ value of 122.81 µg/mL. Additionally, the study quantified total phenolic and flavonoid contents, finding the ethyl acetate fraction to have the highest levels of these compounds. Using 1H−NMR1H−NMR metabolite profiling, 22 metabolites were identified, including notable flavonoids such as quercetin and rutin, believed to contribute to the observed biological activities. Molecular docking analysis further supported the interaction between these metabolites and the α-glucosidase enzyme, indicating their potential role in managing postprandial blood glucose levels. This research provides scientific validation for the ethnomedicinal use of *T. dioica* in diabetes treatment and suggests its potential as a natural therapeutic agent against hyperglycemia.

Aabideen et al. raised concerns regarding the bioactive properties of *Cassia fistula* leaves. The study employed an 80% hydroethanolic extract, which demonstrated significant antioxidant activity and pancreatic lipase (PL) inhibition, with IC50 values of 30.5 µg/mL and 17.31 µg/mL, respectively. Metabolite profiling identified 23 compounds contributing to these activities, including phenolics and flavonoids. Molecular docking studies revealed strong interactions between these compounds and the PL enzyme, suggesting their potential as natural anti-obesity agents. This research highlights the therapeutic potential of *C. fistula* extracts in developing functional foods or phytopharmaceuticals aimed at managing obesity and oxidative stress. Overall, this study supports the use of *C. fistula* as a promising source of natural antioxidants and lipase inhibitors (Contribution 3).

The study by Ardiansyah et al. explored the effects of fermentation on rice bran (RB) from two Indonesian cultivars, Inpari 30 (IPR30) and Cempo Ireng (CI). Rice (*Oryza sativa*) is one of the world’s most important food crops, and is comprised largely of japonica and indica subspecies [4]. This research aimed to analyze the volatile compounds, sensory profiles, total phenolic content (TPC), and antioxidant activity of fermented RB. Using gas chromatography–mass spectrometry, 55 volatile compounds were identified, including alcohols, aldehydes, acids, and ketones, which were significantly altered by fermentation. Sensory analysis revealed that fermentation improved the sensory attributes and acceptance of RB. This study found that fermentation increased TPC and antioxidant activity, with optimal results observed after 72 h. The findings suggest that fermentation enhances the bioactivity and sensory profile of RB, promoting its potential as a functional ingredient for health benefits (Contribution 4).

Suttisansanee et al. focused on the health-promoting properties of various vegetables commonly used in Thai cuisine (Contribution 5). This study emphasizes the significance of phytochemicals found in these vegetables, which are known to combat non-communicable diseases (NCDs) such as diabetes, hypertension, and Alzheimer’s disease [5]. Researchers collected samples from plants vetted by Thailand’s Department of Agriculture and performed phytochemical analyses to assess their antioxidant capacities and inhibitory effects on enzymes linked to NCDs. The key findings revealed that extracts from *Citrus hystrix* and *Senegalia pennata* exhibited potent antioxidant activities, while *Citrus hystrix* also effectively inhibited lipase and angiotensin-converting enzyme, both relevant to obesity and hypertension. Additionally, *Coriandrum sativum* and *Psophocarpus tetragonolobus* showed strong anti-diabetic properties. This study highlights the need for the further exploration of these vegetables to promote healthier dietary practices in Thailand, aiming to reduce the prevalence of NCDs through enhanced consumption of these beneficial plant extracts.

Balčiūnaitė–Murzienė et al. investigated the immunomodulatory effects of the LysM lectin extracted from *Echinacea purpurea* roots (Contribution 6). This experiment was conducted on BALB/c male mice and involved administering purified LysM lectin and a fresh root tincture to assess their impact on immune responses. The results indicated that the LysM lectin significantly increased T lymphocyte counts in the spleen, suggesting its role as an immunostimulant, while the tincture led to a decrease in T lymphocyte levels, indicating immunosuppressive effects. This study highlights the contrasting immune effects of different Echinacea preparations, with the lectin promoting immune activation and the tincture potentially dampening immune responses [6]. This research contributes to understanding the biological activities of *Echinacea purpurea*, particularly its lectins, which have been less studied compared to other components of the plant (Contribution 6).

Ethylene is the key factor involved in the aging and decline of harvested crops, such as cut flowers. Brassinosteroids (BRs), which are natural phytohormones, have been found to variably influence ethylene production and the associated senescence processes in various crops [7]. The study by Darvish et al. explored the effects of 24-epibrassinolide (EBL), a type of brassinosteroid, on the vase life and physiological responses of lisianthus cut flowers. Various concentrations of EBL (0, 3, 6, and 9 µmol/L) were applied to determine their impact on ACC oxidase enzyme activity, which is crucial in ethylene biosynthesis, as well as other physiological factors like chlorophyll and anthocyanin content, malondialdehyde (MDA) production, and water absorption (Contribution 7). The results indicated that a concentration of 3 µmol/L EBL significantly enhanced the vase life of the flowers by reducing ACC oxidase activity and MDA production while promoting chlorophyll and anthocyanin accumulation. Conversely, higher concentrations (6 and 9 µmol/L) negatively affected flower longevity and quality, likely due to increased ACC oxidase activity and ethylene production. The findings suggest that the effects of brassinosteroids on cut flowers are highly concentration-dependent, highlighting the potential for EBL to improve postharvest quality in lisianthus.

Sukhikh et al. focused on the extraction and evaluation of bioactive compounds from *Cotinus coggygria*, a plant with traditional medicinal uses. The researchers optimized extraction conditions using 70% ethanol, a 1:5 hydromodule, for 60 min at 60 °C, which yielded extracts rich in compounds like rutin, quercetin, and ferulic acid. These extracts demonstrated significant antioxidant activity, with a measured value of 145.09 mg AA/g (AA—ascorbic acid), and exhibited antimicrobial properties against pathogens such as *E. coli* and *S. aureus*. The extracts also showed anticancer and antigenotoxic effects, suggesting potential therapeutic applications [8]. This study confirmed that the extracts are safe regarding heavy metals and other contaminants, making them suitable for pharmaceutical exploration. This research highlights the potential of *Cotinus coggygria* as a source of bioactive compounds for health-related applications, providing a scientific basis for its traditional use in medicine (Contribution 8).

Mottaghipisheh analyzed oxypeucedanin (OP), a linear furanocoumarin with significant bioactive properties. Isolated primarily from the *Apiaceae* and *Rutaceae* families, particularly from species like *Angelica* and *Citrus*, OP has been shown to possess antiproliferative, cytotoxic, anti-influenza, and antiallergic effects in preclinical studies. This review details various phytochemical studies that have successfully isolated OP from multiple plant sources using advanced chromatographic techniques. Notably, the methanolic extract of *Angelica dahurica* roots is highlighted as the richest natural source of OP. This publication emphasizes the importance of exploring OP and its derivatives as potential drug candidates for further biological assessments [9]. The findings underscore the relevance of furanocoumarins in pharmacognosy and their traditional medicinal applications, suggesting a promising avenue for future research into their therapeutic potential (Contribution 9).

*Dioscorea* (Yam) species are rich in nutritional and pharmacological value due to their content of polysaccharides, steroidal saponins, allantoin, and polyphenols like flavonoids [10]. Kim et al. investigated the metabolite composition of yam leaves, which are often discarded despite their potential health benefits. Researchers conducted a comprehensive analysis of two yam species, Danma and Dunggeunma, using advanced mass spectrometry techniques to profile metabolites and assess antioxidant activity across different harvest times. This study found that while primary metabolites like amino acids and sugars decreased over time, secondary metabolites such as flavonoids and phenanthrenes increased, particularly in leaves harvested in August, which exhibited the highest antioxidant activity. The analysis revealed significant differences in metabolite profiles between the two yam species, with certain compounds correlating positively with antioxidant capacity. Notably, secondary metabolites were found to be more abundant in the leaves than in the tubers, suggesting that yam leaves could serve as a valuable source of bioactive compounds. The findings advocate for the utilization of yam leaves as a sustainable resource for nutraceutical and pharmaceutical applications, emphasizing their potential to mitigate agricultural waste and contribute to environmental sustainability (Contribution 10).

The study of Krizhanovska et al. highlighted the impact of cultivation on the phytochemical properties and biological activities of greater celandine (*Chelidonium majus*) (Contribution 11). This study compared extracts from wild-grown and cultivated plants, revealing that cultivation significantly enhances the total alkaloid content, particularly increasing the levels of chelidonine, which became the dominant alkaloid in cultivated specimens. Using LC/MS techniques, the research identified twelve alkaloids and ten flavonol glycoconjugates in the extracts, with no significant differences in flavonoid content between the two groups. Cytotoxicity assays demonstrated that extracts from cultivated plants exhibited higher cytotoxic effects against cancer cell lines B16-F10, HepG2, and CaCo-2, with the strongest effect observed in B16-F10 cells. The findings suggest that cultivation practices not only improve the yield of bioactive compounds but also enhance the therapeutic potential of *C. majus*, supporting its use in traditional medicine [11].

The research conducted by Vilkickyte and Raudone identifies 43 phenolic and triterpenoid compounds in the leaves of *Vaccinium vitis-idaea* L., with the highest concentrations observed during autumn and early spring (Contribution 12). The findings highlight significant correlations between the levels of these compounds and environmental factors such as temperature, sunshine duration, and soil macronutrients. This study reveals that the accumulation of these secondary metabolites is influenced by both phenological stages and geographical conditions, suggesting that harsher weather may enhance the biosynthesis of beneficial compounds. The researchers utilized HPLC-PDA methods for quantitative and qualitative analysis, demonstrating that the chemical composition of lingonberry leaves varies significantly throughout the year and across different locations. This research emphasizes the potential of lingonberry leaves as a valuable source of nutraceuticals, given their higher biological activity compared to fruits, and underscores the importance of understanding environmental influences on plant metabolism for optimizing the harvesting of these compounds [12].

Kupe et al. examined the phenolic content and antioxidant properties of various parts of nine clones of the ‘Karaerik’ grape, a prominent cultivar in Turkey (Contribution 13). This study employed the Folin–Ciocalteu method to quantify total phenolic content, alongside three antioxidant assays: DPPH, FRAP, and TEAC. The results indicated that grape seeds consistently exhibited the highest levels of phenolic compounds and antioxidant activity across all clones, followed by the peel and pulp. Among the clones, Clone 8 had the highest total phenolic content at 149 mg GAE/100 g FW, while Clone 3 had the lowest at 111 mg GAE/100 g FW. This research highlights the rich presence of beneficial phenolic compounds such as gallic acid and quercetin in grape seeds, suggesting their potential for health benefits. These findings underscore the importance of clonal selection in viticulture, indicating that certain clones may be more advantageous for both grape breeders and health-conscious consumers due to their superior antioxidant properties [13].

Raudone et al. investigated the phytochemical profiles and antioxidant properties of various *Achillea* species, which are underutilized wild plants in Turkey (Contribution 14). Researchers collected plant samples from different sites and utilized HPLC-PDA methods to identify and quantify phenolic compounds, particularly focusing on hydroxycinnamic acids and flavonoids. The findings revealed significant species-specific variations in the distribution of these compounds across different plant parts, with *A. setacea* exhibiting the highest total amounts of caffeoylquinic acids, while *A. arabica* showed notable levels of flavonoids, particularly quercetin and luteolin derivatives. The antioxidant activity was assessed through radical scavenging assays, indicating that the leaves of all studied species had superior antioxidant capacity compared to inflorescences and stems. This study provides essential insights into the chemical diversity of *Achillea* species, highlighting their potential for further research and development in pharmaceuticals and natural products [14].

Kupnik et al. analyzed various extraction techniques to recover bioactive compounds from pomegranate peels, a significant agricultural waste (Contribution 15). The researchers employed methods such as Cold Maceration, Ultrasonic Extraction, Soxhlet Extraction, and Supercritical Fluid Extraction (SFE) using different solvents, including methanol, ethanol, and acetone, while varying the operating pressure in SFE. The results indicated that SFE at 20 MPa yielded the highest total polyphenol content, particularly ellagic acid, demonstrating the effectiveness of supercritical extraction in recovering valuable compounds. Additionally, this study quantified the antimicrobial activity of the extracts against several microorganisms, identifying a Minimal Inhibitory Concentration (MIC) of 2.7 mg/mL for SFE extracts against various bacteria. These findings highlight the potential of pomegranate peels as a source of high-value bioactive compounds, suggesting that these waste materials can be transformed into functional products with health benefits [15].

The study conducted by Mohammed investigates the phytochemical properties, antioxidant potential, and cytotoxicity of *Artemisia absinthium* (wormwood) from the central region of Saudi Arabia (Contribution 16). Utilizing gas chromatography–mass spectrometry (GC-MS) and gas chromatography with flame ionization detection (GC-FID), this research identified 34 volatile compounds, with cis-davanone being the most prevalent at 52.51%. The essential oil exhibited significant antioxidant activity, measured through various assays, including total antioxidant capacity (TAC) and DPPH scavenging activity, indicating its potential health benefits. This study also assessed the cytotoxic effects of the essential oil, revealing a limited impact on various cell lines, suggesting the plant’s safety as an edible species. Notably, this research highlights how environmental conditions influence the chemical composition of *A. absinthium*, underscoring the importance of local growing conditions on the plant’s volatile oil quality [16].

Dranseikienė et al. highlighted the potential of cyano-phycocyanin (C-PC), a pigment derived from the cyanobacterium *Arthrospira platensis* (Spirulina), for treating various skin conditions (Contribution 17). This review highlights C-PC’s multifaceted biological activities, including wound healing, antimicrobial, antioxidative, anti-inflammatory, antimelanogenic, and anticancer properties. The authors discuss how C-PC can enhance fibroblast proliferation and migration, crucial for wound healing, and demonstrate its effectiveness in both in vitro and in vivo models. Additionally, C-PC exhibits significant antimicrobial activity against several bacterial strains, presenting a promising alternative to conventional antibiotics, especially in the face of rising drug resistance. This review emphasizes the need for further research into the biotechnological applications and pharmaceutical formulation requirements of C-PC for effective topical treatments [17].

In conclusion, these findings underscore the importance of exploring natural compounds for their health benefits, advocating for sustainable cultivation and utilization practices. This research highlights the potential of these bioactive compounds in developing nutraceuticals and pharmaceuticals, contributing to the advancement of public health nutrition and sustainable agriculture. Overall, the studies provide a comprehensive understanding of the therapeutic potential of plant-based bioactives, encouraging further exploration and application in various health-related fields. These investigations highlight the vast potential of plant-based compounds in enhancing health and wellness while also promoting sustainable and environmentally friendly practices.
